# Characteristic impairment of progesterone response in cultured cervical fibroblasts obtained from patients with refractory cervical insufficiency

**DOI:** 10.1038/s41598-023-37732-7

**Published:** 2023-07-20

**Authors:** Yosuke Sugita, Yoshimitsu Kuwabara, Akira Katayama, Shigeru Matsuda, Ichiro Manabe, Shunji Suzuki, Yumiko Oishi

**Affiliations:** 1 Department of Biochemistry and Molecular Biology, Nippon Medical School, 1-1-5 Sendagi, Bunkyo-ku, Tokyo 113-8602 Japan; 2 Department of Obstetrics and Gynecology, Nippon Medical School, 1-1-5 Sendagi, Bunkyo-ku, Tokyo 113-8602 Japan; 3 Department of Systems Medicine, Chiba University Graduate School of Medicine, 1-8-1 Inohana, Chuo-ku, Chiba 260-8670 Japan

**Keywords:** Molecular biology, Reproductive disorders

## Abstract

Preterm birth (PTB) is the leading cause of neonatal mortality, and reducing the PTB rate is one of the most critical issues in perinatal medicine. Cervical insufficiency (CI), a major cause of PTB, is characterised by premature cervical ripening in the second trimester, followed by recurrent pregnancy loss. Although multiple clinical trials have suggested that progesterone inhibits cervical ripening, no studies have focused on progesterone-induced molecular signalling in CI. Here, we established a primary culture system for human uterine cervical fibroblasts using a sample of patients with refractory innate CI who underwent transabdominal cervical cerclage and patients with low Bishop scores who underwent elective caesarean section as controls. RNA sequencing showed that the progesterone response observed in the control group was impaired in the CI group. This was consistent with the finding that progesterone receptor expression was markedly downregulated in CI. Furthermore, the inhibitory effect of progesterone on lipopolysaccharide-induced inflammatory stimuli was also impaired in CI. These results suggest that abnormal cervical ripening in CI is caused by the downregulation of progesterone signalling at the receptor level, and provide a novel insight into the molecular mechanism of PTB.

## Introduction

Preterm birth (PTB) is the leading cause of neonatal mortality and considerably impairs perinatal outcomes. PTB is increasing worldwide, with approximately 15 million premature babies born annually and grobal PTB rates of 5–18%^1^. Thus, reducing the PTB rate is one of the most critical issues in perinatal medicine.

The major causes of PTB are infection^2–4^, maternal-foetal stress^5–8^, chorionic-decidual bleeding^9–11^, uterine hyperextension^12, 13^, and premature cervical ripening^14–16^. These factors are involved in pathogenesis of PTB in a complex manner. Cervical insufficiency (CI) is defined as “the inability of the uterine cervix to retain a pregnancy in the second trimester in the absence of clinical contractions, labour, or both”^17^. It is characterised by premature cervical ripening and prolapse of the membrane, which is followed by recurrent pregnancy loss in the second trimester without clinical uterine contractions and is distinguished from infection-induced PTB. The diagnosis of CI is made based on pregnancy history and ultrasound findings, and cervical cerclage is performed to mechanically support the cervix to reduce the risk of PTB^17^. CI is likely to develop after cervical surgery^18^; however, some patients do not have a history of surgery. Such conditions are thought to constitute innate abnormalities; however, the underlying molecular mechanisms are unknown.

The cervix is composed of fibroblasts and an extracellular matrix consisting mainly of collagen. Normally, the cervix is tightly closed during most of the gestational period to maintain the uterine contents. Cervical ripening is induced before the onset of labour, tissue swelling and decreasing collagen density provide softening and ripening of the cervix, and the cervix opens as a passageway for the foetus^15, 19^. Innate CI can be regarded as a pathological condition in which cervical ripening occurs early for some reason.

There are refractory instances in which PTB cannot be prevented even with conventional transvaginal cervical suturing in both innate cases and those after cervical surgery. Transabdominal cervicoisthmic cerclage (TAC) is an effective treatment to maintain pregnancy in both those cases^20, 21^. When following up on innate CI cases that underwent TAC, we found that the cervix maintained a tight and firm structure during early pregnancy and showed rapid ripening changes in the second trimester, similar to those typically observed in the third trimester. Pregnancies were then maintained until term with a fully mature cervix by suturing at the isthmus of the cervix.

In both humans and rodents, the administration of progesterone (P4) receptor inhibitors induces cervical ripening and leads to parturition^1, 14, 22^. Multiple clinical trials have demonstrated the usefulness of transvaginal progesterone supplementation in patients with a shortened cervical length, suggesting that progesterone has an inhibitory effect on cervical ripening. Cervical ripening is an inflammation-like process synergistically induced by inflammatory cytokines and proteases secreted by cervical fibroblasts and locally infiltrating immune cells^23^. Using a primary culture system of human cervical fibroblasts, we previously reported that progesterone suppressed the inflammatory response induced by lipopolysaccharides (LPS)^24^. Because most progesterone receptors are localised in stromal fibroblasts^14, 25^, progesterone is presumed to maintain pregnancy by controlling the inflammatory response in the endocervical stroma.

As many spontaneous PTBs are preceded by cervical ripening^15^, elucidating the mechanism of early cervical ripening in refractory CI, which can be regarded as the extreme type, is considered an important prerequisite to understanding the pathology of PTB. In this study, we established a fibroblast culture system from the cervical tissues of patients with innate refractory CI and focused on changes in the responsiveness to P4, aiming to elucidate the molecular pathogenesis of this condition.

## Results

### Cervical tissue sampling

Cervical tissue samples were collected from pregnant women with refractory cervical insufficiency (CI) characterized by a matured cervix (n = 3) and from pregnant women without cervical maturity as controls (n = 3). CI was defined as a pregnancy history of miscarriage preceded by asymptomatic cervical dilation and prolapse of membrane in the second trimester. The current pregnancies of the patients were maintained until 35 weeks of gestation with TAC. Controls included patients with no history of mid-term miscarriages or PTB. They had undergone an elective caesarean section due to their pelvic position or previous caesarean section before labour with an immature cervix (Bishop score < 4 points). After conization, patients with bacterial vaginitis, clinical chorioamnionitis, hypertensive disorder, and/or gestational diabetes mellitus were excluded from the study. Bacterial vaginitis was diagnosed on the basis of the Nugent score. A breakdown of these cases is shown in Table 1.

**Table 1:** Background of patients.

	Case	Control_1	Control_2	Control_3	CI_1	CI_2	CI_3
	Age	38	35	32	44	32	35
	Gravida	3	1	2	3	3	4
	Parity	1	0	1	0	0	1
	Surgical history	CS	Myomectomy	CS	None	None	CS
This pregnancy	GW at CS	38	38	38	37	37	35
	Cerclage	None	None	None	TAC	TAC	TAC
	BV	None	None	None	None	Intermediate	None
	CAM	Untested	Untested	Untested	BlancI	Untested	BlancI
	Other complications	None	None	None	None	None	None
Previous pregnancy	History of second trimester pregnancy loss	None	None	None	21w pPROM	21w prolapse of membrane	18w pPROM
					13w prolapse of membrane	21w prolapse of membrane (after vaginal cerclage)	19w prolapse of membrane
	History of PTL	None	None	None	None	None	None

*GW* gestational week, *CS* caesarean section, *PTL* preterm labour, *BV* bacterial vaginosis, *CAM* chorioamnionitis, *pPROM* preterm Premature Rupture of Membranes, *TAC* transabdominal cervical cerclage.

### Comprehensive analysis of gene expression response to P4 addition

To investigate the gene expression change induced by P4, we performed whole-transcriptome analysis using RNA sequencing (RNA-seq). Uterine cervical fibroblasts (UCFs) derived from the control (n = 2) and CI (n = 2) were precultured with 10 nmol/L beta-oestradiol in duplicate for 24 h and subsequently incubated with progesterone (1 μM) or ethanol alcohol (solvent) alone for 6 h. RNA was extracted from each sample and subjected to RNA-seq analysis. Gene set enrichment analysis (GSEA) of Molecular Signatures Database (MSigDB) hallmark gene sets (Table 2) showed no trend leading to pathophysiology when comparing control and CI under conditions without P4. In control-derived UCFs, P4 administration enhanced the expression of the gene set associated with sex hormone response and suppressed the proinflammatory gene set, including TNFα signalling via NFκB, inflammatory response, and interferon response. In contrast, in CI-derived UCFs, P4 administration did not change the expression of the gene set associated with sex hormone response but enhanced the expression of the proinflammatory gene set, including TNFα signalling and interferon response. Additionally, P4 administration produced no significant trend in the extracted gene sets.

**Table 2:** Comparison of gene expression by Hallmarks Gene Set Enrichment Analysis.

Upregulated in control		Upregulated in CI	
Gene set	*p* value	Gene set	*p* value
PROTEIN_SECRETION	0.001	E2F_TARGETS	< 0.001
MYOGENESIS	0.011	G2M_CHECKPOINT	< 0.001
KRAS_SIGNALING_UP	0.011	MYC_TARGETS_V1	< 0.001
EPITHELIAL_MESENCHYMAL_TRANSITION	0.011	MYC_TARGETS_V2	< 0.001
TGF_BETA_SIGNALING	0.013	UNFOLDED_PROTEIN_RESPONSE	< 0.001
APICAL_JUNCTION	0.018	SPERMATOGENESIS	< 0.001
ANDROGEN_RESPONSE	0.018	DNA_REPAIR	< 0.001
ANGIOGENESIS	0.021	MTORC1_SIGNALING	< 0.001

Upregulated by P4 in control		Downregulated by P4 in control	
Gene set	*p* val	Gene set	*p* val
E2F_TARGETS	< 0.001	CHOLESTEROL_HOMEOSTASIS	< 0.001
G2M_CHECKPOINT	< 0.001	MTORC1_SIGNALING	< 0.001
MYC_TARGETS_V1	0.003	TNFA_SIGNALING_VIA_NFKB	< 0.001
ANDROGEN_RESPONSE	0.005	UV_RESPONSE_DN	< 0.001
ESTROGEN_RESPONSE_LATE	0.020	HYPOXIA	0.007
KRAS_SIGNALING_DN	0.034	INTERFERON_GAMMA_RESPONSE	0.009
		KRAS_SIGNALING_UP	0.013
		INFLAMMATORY_RESPONSE	0.020

Upregulated by P4 in CI		Downregulated by P4 in CI	
Gene set	*p* value	Gene set	*p* value
SPERMATOGENESIS	< 0.001	MYC_TARGETS_V1	< 0.001
MITOTIC_SPINDLE	< 0.001	OXIDATIVE_PHOSPHORYLATION	< 0.001
TNFA_SIGNALING_VIA_NFKB	0.009	EPITHELIAL_MESENCHYMAL_TRANSITION	< 0.001
KRAS_SIGNALING_UP	0.011	COAGULATION	< 0.001
INTERFERON_GAMMA_RESPONSE	0.013	HYPOXIA	< 0.001
INTERFERON_ALPHA_RESPONSE	0.026	APOPTOSIS	< 0.001
KRAS_SIGNALING_DN	0.033	UNFOLDED_PROTEIN_RESPONSE	< 0.001
		REACTIVE_OXYGEN_SPECIES_PATHWAY	< 0.001

Gene sets differentially expressed between control and CI in no treatment and up or downregulated gene sets by P4 in control, and CI are shown. Statistical significance was set at *p* < 0.05, and the top eight gene sets are shown.

**Figure 1:**
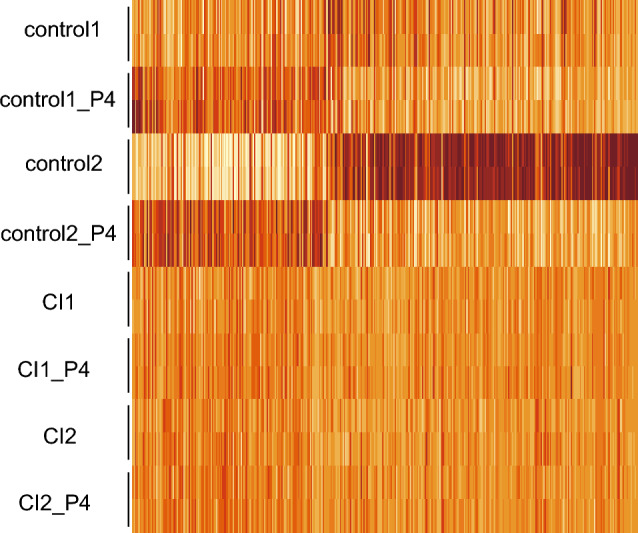
Comparison of expression levels of P4 response genes using RNA sequencing. Heatmap showing the level of expression of genes whose expression levels were changed (nominal *p* < 0.05) upon P4 treatment in either the control or CI groups.

Next, a heat map was created based on the expression levels of 387 genes whose expressions were changed by P4 administration (*p* < 0.05). The entire list of 387genes, consisting of 150 upregulated and 237 downregulated genes, can be found in Supplementary Tables S1 and S2. Both up- and downregulated control-derived UCFs showed a common trend of expression change in response to P4, whereas no response to P4 was observed in CI-derived UCFs (Fig. 1).

### Comparative analysis of P4 response focusing on specific molecules

The RNA sequencing analyses demonstrated that contrary to our initial expectation, the molecular groups reported to be involved in prior cervical ripening, including Has2^26, 27^, VCAN, THBS1, COL5A1, and MYOCD^28^, did not show significant gene expression changes between cells obtained from control and CI patients in response to P4 stimulation. This finding was also confirmed by the real-time PCR analyses (Supplementary Fig. 2).

**Figure 2:**
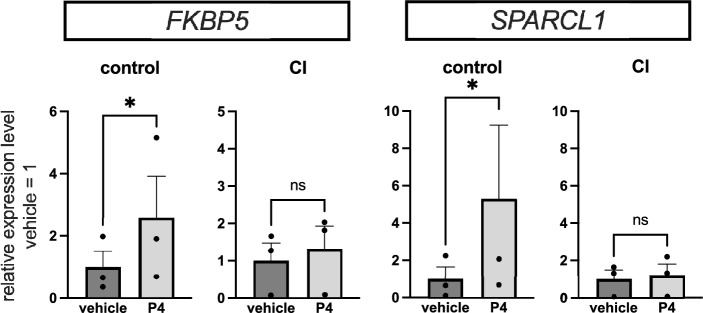
RT-PCR results of expression changes upon P4 administration to UCFs. Reverse transcribed cDNA was used from RNA extracted from the control (n = 3) and CI (n = 3) groups cultured under their respective conditions. The relative expression level of the P4 response gene 6 h after P4 administration using UCFs of the control and CI (mean of control = 1) groups. Data are expressed as the mean ± SEM and analysed using a ratio paired t-test. * indicates *p* < 0.05.

Among the upregulated genes as determined by RNA-seq analysis, *FKBP5* (FKBP Prolyl Isomerase 5)^29, 30^ and *SPARCL1* (SPARC-Like Protein 1)^31, 32^ are representative P4-responsive genes aligned with previous reports. We designed primers for *FKBP5*, a glucocorticoid receptor co-chaperone, and *SPARCL1*, a secreted glycoprotein that constitutes the extracellular matrix. P4 (1 μM) dissolved in ethanol or ethanol as a control was added to the UCFs culture system, RNA was extracted 6 h later, and an inverted-copy reverse transcriptase RT-PCR was used to analyse the complementary cDNA. FKBP5 and SPARCL1 expression level was significantly upregulated in controls following P4 treatment (*p* < 0.05) while it remained unaffected in the CI-derived cells (Fig. 2).

### Analysis of progesterone receptor expression

**Figure 3:**
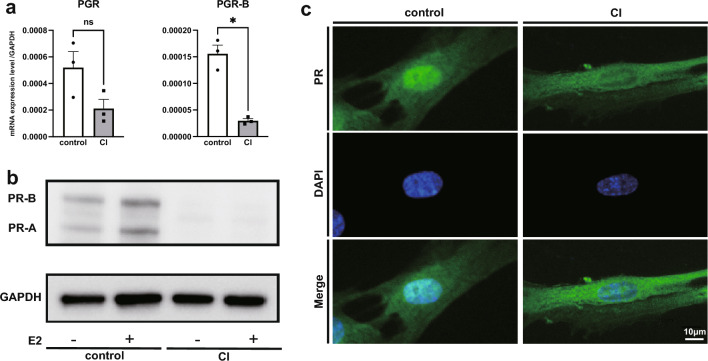
Comparison of PR expression in the control and CI groups. (**A**) Quantitative RT-PCR using UCFs of the control and CI groups incubated in the presence of beta-oestradiol (10 nmol//L) for 24 h. Data are expressed as the mean ± SEM and analysed using the ratio paired t-test. *Indicates *p* < 0.05. (**B**) Western blotting using 10 μg of protein extracted from cells cultured without or with beta-oestradiol (10 nmol//L) PR-A:90 kDa, PR-B:118 kDa. Images of PR and GAPDH staining were obtained using the same lane of the same gel, with an exposure time of 300 s for PR and 60 s for GAPDH. (**C**) Immunocytochemistry images of UCFs cultured for 24 h with beta-oestradiol (10 nmol/L) using the anti-PR-A/B and anti-DAPI antibodies for nuclear staining. Scale bars: 10 μm.

Next, we focused on the differences in progesterone receptor (PR) expression levels in fibroblasts as a possible mechanism for the differential P4 response. According to the results of RNA sequence analysis, P4 addition did not change the PR expression level in either CI-derived or control-derived UCFs. Cell immunostaining also showed no difference in PR expression due to the addition of P4 (data not shown). Therefore, a comparative analysis of PR expression was performed by real-time PCR, western blotting, and cell immunostaining, using CI-derived and control-derived UCFs treated with P4. After 24 h of incubation with E2, cells were harvested for comparative analysis of the expression of the two isoforms of PR, PR-A and PR-B. Real-time PCR revealed no significant differences in the gene expression of the progesterone receptor gene (*PGR*); however, a significant (*p* < 0.05) decrease in the expression of *PGR-B*, an isoform with a more extended transcription region, was observed in the CI group compared to that in the control group (Fig. 3A). Western blotting analysis of protein expression levels also showed no bands corresponding to PR-A and PR-B in CI (Fig. 3B). The photograph of the full-length gel is shown in Supplementary Fig. S1. In immunocytochemistry, the PR was stained in the nuclei of the control group and not in the nuclei of the CI group (Fig. 3C). The comparably weak cytoplasmic staining in both CI-derived and control-derived UCFs can be regarded as a non-specific reaction, based on the western blot analysis showing non-specific bands in both UCFs conducted using the same antibody.

### Analysis of inflammatory response to LPS

**Figure 4:**
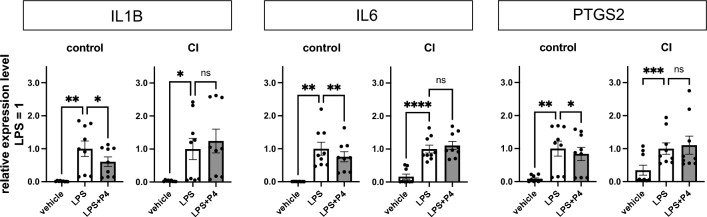
RT-PCR results demonstrating the suppressive effect of P4 on LPS-induced inflammation. Reverse-transcribed cDNA was obtained from RNA extracted from the control group (n = 3) and the CI group (n = 3), both cultured under their respective conditions. The relative expression level of proinflammatory cytokines was assessed 6 h after pre-administration of P4, followed by LPS treatment 1 h prior (with the mean expression level of the LPS alone treatment set as 1). The graph shows results from triplicate samples for each group. Data are presented as the mean ± SEM and analysed using one-way ANOVA with Tukey’s post-hoc test. *Indicates *p* < 0.05.

To investigate the effect of differential P4 signalling on the inflammatory response, next used real-time PCR to quantitatively analyse the inhibitory effect of P4 (1 µM) on the inflammatory response to LPS, which are exogenous ligands. UCFs were treated with LPS (2 µg/mL) for 1 h, then P4 (1 μM) dissolved in ethanol or ethanol as a control was added for 6 h, RNA was extracted, and cDNA was reverse-transcribed and analysed using RT-PCR. The suppressive effect of P4 on the induction of *IL-1B*, *IL-6*, and prostaglandin-endoperoxide synthase 2 (*PTGS2*) expression by LPS was observed in the control group but not in the CI group (Fig. 4). This suggests that the P4 signalling-mediated suppressive effect on the inflammatory response to LPS is impaired in the cervical tissues of CI.

## Discussion

In this study, the response to P4 administration observed in cultured UCFs derived from pregnant women with an unripened cervix was completely diminished in those UCFs derived from patients with refractory CI. We further confirmed that a pronounced decrease in the expression of progesterone receptors accompanied this loss of response to P4 administration. This explains the putative molecular mechanism underlying refractory endogenous CI, which is also consistent with the concept that human cervical ripening is caused by the withdrawal of P4 signalling.

Obtaining fully ripened cervical tissue unaffected by labour and/or delivery is difficult, and the existing study used cervical tissue obtained immediately after parturition. With this study design, it is impossible to assess the molecular change accompanied by cervical ripening itself, because mechanical stress to the cervix due to cervical dilatation and the systemic maternal change associated with labour pains may affect the molecular expression. In the present study, the unique clinical sample from a ripened cervix of patients who underwent TAC enabled us for the first time to investigate pure molecular changes associated with cervical ripening while excluding the effects of delivery for the first time.

P4 is essential for maintaining pregnancy, and the administration of PR antagonists is known to induce rapid cervical ripening. There are two isoforms of PR, PR-A and PR-B. The PR-B signal primarily contributes to the maintenance of pregnancy, and its function is regulated by the expression balance of PR-A^33–35^. In this study, UCFs cultures derived from patients with innate refractory CI showed a marked decrease in PR expression at the protein level and a more pronounced downregulation of the functional receptor PR-B at the mRNA level. As indicated in the heatmap, the overall progesterone response in CI was significantly attenuated. However, as shown in Table 2, partial responses were observed in CI. It has been reported that progesterone can also signal through the glucocorticoid receptor (GR). We found no significant difference in GR expression between the control and CI (Supplementary Fig. 3). Therefore, while the response mediated by PR was diminished, we speculate that residual partial progesterone response may be attributable to crosstalk mediated by GR. In a control UCFs culture system established from the immature cervix of full-term pregnant women, genes responsive to P4 administration were found to be similar to those in a previous comprehensive expression analysis report using non-pregnancy cervical tissue^36^.

According to GSEA, the functional roles of these genes are involved in the suppression of inflammatory cytokines, which is consistent with the results of previous studies that focused on P4 signalling in cervical tissue^24^. As these genes are considered responsible for the maintenance of pregnancy via PR signalling, they might be important for elucidating the mechanisms of cervical ripening and constitute candidates for new therapeutic targets and/or biomarkers for predicting the risk of PTB.

In this study, we found that cases of refractory CI showed acute cervical ripening in the second trimester.

We further analysed the UCFs established from the cervical stroma and found that those from patients with refractory innate CI lost a major part of PR expression and were characterized by an impaired P4 response. These findings strongly suggest that an impaired P4 response caused by decreased PR expression is involved in some refractory cases of endogenous CI. We assume that in such cases, the expression of PR in cervical stromal cells will be suppressed during mid-gestation, resulting in rapid maturation and leading to exposure and rupture of the foetal membranes.

At present, it is unclear what causes such differences in PR expression levels. ER signalling is known as an upstream factor that promotes PR expression. All experiments in this study were performed using UCF precultured for 24 h in media containing beta-oestradiol in the expectance of inducing stable PR expression. According to the real-time PCR analyses, no significant differences in ER expression were observed between control and CI cells (Supplementary Fig. 3). Considering that large amounts of oestrogen are constantly produced by the placenta during pregnancy, it is difficult to explain differences in PR expression levels in relation to ER signalling.

As this unique property was maintained even after several subcultures, it is reasonable to assume that it is based on inherited genetic or epigenetic factors. As there were no problems with the establishment of pregnancy or its course until the first trimester, congenital PR deficiency may not be involved. It appears that these phenomena are caused by a specific unknown mechanism during the second trimester of pregnancy when premature cervical ripening becomes apparent. Exploring this mechanism will provide data for understanding how the trigger of P4 withdrawal that precedes parturition is regulated in both pre- and full term instances. As a next step, we plan to conduct an integrated omics analysis using whole genome sequence data from patients with endogenous CI and epigenome data (by ChIP- or ATAC-sequence) from each sample, in addition to elucidating the RNA sequence data.

From a clinical perspective, preventive P4 therapy, which is widely used for pregnant women at high risk of premature birth, is considered partially effective or ineffective for patients with refractory innate CI. There are currently no studies investigating the therapeutic effects of progesterone focused on refractory CI patients, which constitutes an important topic for future investigations. Also, further investigation is needed to determine whether this putative pathophysiological mechanism characterized by loss of PR expression can be applied to cervical ripening observed prior to other types of PTB and/or term delivery.

The study was subject to some limitations. First, we used a small number of cases, as we targeted the rare instances of patients with refractory endogenous CI who underwent TAC. Second, because of the nature of the experiment using the primary culture system, the number of samples that could be used for each experiment was limited to avoid property changes due to passage. Increasing the number of cases to verify the generalizability and validity of the study results is challenging due to the inclusion of the invasive cervical tissue biopsy procedure. In the future, the use of non-invasive methods such as endocervical scraping samples and/or cervical mucus collected during pregnancy could be valuable for indirectly assessing changes in PR expression. By analysing the P4-responsive molecules identified in these samples, it would be possible to evaluate PR expression.

In conclusion, the impaired P4 response accompanied by decreased PR expression was characteristic of cultured cervical fibroblasts obtained from patients with refractory CI. Further investigations targeting sensitive P4-responsive molecules and elucidating the regulatory mechanisms of PR expression are essential for the development of new clinical strategies for the prevention of PTB.

## Methods

### Establishment of primary UCFs culture

As previously reported, we prepared UCFs from uterine tissues^23^. All samples were collected with written informed consent obtained from the subjects. After delivery of the foetus and placenta in a scheduled caesarean section before the onset of labour, we identified the closed endocervix through the uterine wound and collected 5 mm of cervical tissue. The study protocol was approved by the Nippon Medical School Hospital Ethics Committee (approval number: R1-08–1176) and was carried out in accordance with the Ethical Guidelines for Medical and Biological Research Involving Human Subjects. The uterine cervical tissue was cut into small pieces and incubated in Dulbecco’s modified Eagle’s medium (Gibco/Invitrogen, Carlsbad, CA, USA) containing 10% foetal bovine serum (HyClone, Logan, UT, USA) in a humidified atmosphere of 5% CO_2_ and 95% air at 37 °C. The culture medium was changed twice per week. Once 80% confluent, fibroblasts were enzymatically detached with 0.25% trypsin (Gibco) and cultured in 10 cm culture plates. All experiments were performed using cells from passages 4 to 8. UCFs were precultured for 24 h in media containing 10 nmol/L beta-oestradiol conforming to the physiological conditions present during pregnancy and subsequently incubated in the presence or absence of P4 and LPS isolated from *Escherichia coli* (L2880, Sigma-Aldrich, St. Louis, MO, USA).

### RNA sequencing

Total RNA was extracted from cells and purified using an RNeasy Mini Kit (Qiagen, Hilden, Germany). Sequencing libraries were prepared from the polyA-enriched mRNA as follows: First, poly A-enriched mRNA was fragmented in 2 × RNA binding buffer with Oligo d(T)25 Magnetic Beads (New England BioLabs, Ipswich, MA, USA) and incubated at 65 °C for 5 min. They were immediately chilled on ice prior to the next step. Thereafter, first-strand synthesis reaction buffer (4.8 µL) and random primers (1.2 µL; New England BioLabs) were added to the washed beads and heated at 94 °C for 3 min. After incubation, water (8 µL) and NEBNext First Strand Synthesis Enzyme Mix (2 µL; New England BioLabs) were added and incubated in a PCR machine under the following conditions: 25 °C for 10 min, 42 °C for 15 min, 70 °C for 15 min, and a 4 °C hold. Thereafter, water (48 µL), second synthesis reaction B (8 µL), and second Strand Synthesis Enzyme Mix (4 µL) were added and incubated at 16 °C for 60 min with a 4 °C hold. The product was subsequently purified using AMPureXP (Beckman Coulter Inc., Brea, CA, USA) beads according to the manufacturer's instructions and eluted with 10 mM Tris–HCl (26 µL; pH 8.0). Thereafter, purified cDNA (25 μL) was subjected to end prep and adaptor ligation using the NEBNext Ultra II End Repair/dA-Tailing Module (New England BioLabs) and NEBNext Ultra II Ligation Module (New England BioLabs) according to the manufacturer's instructions. Libraries were PCR-amplified for 7–15 cycles, sizes selected using gel extraction, quantified using a Qubit dsDNA HS Assay Kit (Thermo Fisher Scientific, Waltham, MA, USA), and sequenced on either a Genome Analyzer II (Illumina Inc., San Diego, CA, USA) or Hi-Seq 2000 (Illumina Inc.) for 51 cycles.

### Reverse-transcription PCR

RNA was extracted using an RNeasy Mini Kit (Qiagen). Total RNA (2 mg) was reverse-transcribed for subsequent PCR analysis. Real-time PCR was performed in a final reaction volume of 20 mL, containing 2 × Power SYBR Green PCR Master Mix (10 mL; Applied Biosystems, Waltham, MA, USA), 10 µM forward and reverse primer (0.5 mL), DNA template (4 mL), and RNase-free water (5 mL). The primer pairs for each gene were as follows: glyceraldehyde-3-phosphate dehydrogenase (GAPDH), (forward) 5′- TCGACAGTCAGCCGCATCT -3′, (reverse) 5′- CTAGCCTAAAGGGTTTCTCT -3′, progesterone receptor (PGR), (forward) 5′- AGGTCTACCCGCCCTATCTC -3′, (reverse) 5′- GTTGTGCTGCCCTTCCATTG-3′, progesterone receptor B(PGR-B) (forward) 5′- TATCTCCCTGGACGGGCTAC-3′, (reverse) 5′- TGTCCAAGACACTGTCCAGC -3′, FKBP prolyl isomerase 5 (FKBP5), (forward) 5′- AAGAGCTTCGAAAAGGCCAAAG -3′, (reverse) 5′- TGAATCACCGCCTGCATGT -3′, SPARC like 1 (SPARCL1), (forward) 5′-CGGTAGCACCTGACAACACT -3′, (reverse) 5′-TTTTTCAGCCTTATGGTGGGA -3′, IL1B, (forward) 5′ − CAGAAGTACCTGAGCTCGCC -3′, (reverse) 5′-AGATTCGTAGCTGGATGCCG -3′, IL6, (forward) 5′ − CATCCTCGACGGCATCTCAG -3′, (reverse) 5′- TCACCAGGCAAGTCTCCTCA -3’, prostaglandin-endoperoxide synthase 2 (PTGS2), (forward) 5′ − CCCTTCTGCCTGACACCTTT -3′, and (reverse) 5′- TTCTGTACTGCGGGTGGAAC -3’. PCR was performed in MicroAmp Optical 96-well plates with optical adhesive covers (Applied Biosystems). Amplification and detection were performed using the StepOnePlus system (Applied Biosystems). The amplification conditions consisted of pre-incubation at 95 °C for 20 s, 40 cycles of denaturation at 95 °C for 3 s, and annealing at 60 °C for 30 s, followed by a melting curve analysis to confirm that the target amplicons were detected. The fluorescence data were analysed using StepOne Software. The samples were assayed in triplicate for each gene and the mean expression was used for subsequent analyses. GAPDH expression was used to normalise the mRNA levels in each sample. Relative expression was calculated using the ∆∆Ct method.

### Immunocytochemistry

To confirm PR expression, the cells were incubated in the presence of 10 nmol/L beta-oestradiol for 24 h. The cells were fixed with 4% paraformaldehyde for 10 min, washed with phosphate-buffered saline (PBS), and permeabilised with 0.1% Triton-X100 in PBS for 10 min. The cells were subsequently blocked with a solution of 10% goat serum and 1% bovine serum albumin (Sigma-Aldrich) in PBS for 60 min before incubation with a rabbit monoclonal antibody against human PR A/B (D8Q2J, Cell Signalling Technology, Danvers, MA, USA; 1:500 dilution) in Can Get Signal Immunostain Solution A (Toyobo, Osaka, Japan) for 12 h at 4 °C. The cells were then washed with PBS and incubated with Alexa Fluor 488 AffiniPure Goat Anti-Rabbit IgG antibody (Jackson ImmunoResearch Laboratories, West Grove, PA, USA; 1:2000 dilution) in Can Get Signal Immunostain Solution B for 60 min at 20–25 °C. To visualize the cellular distribution of PRs, nuclei were labelled by rewashing the slides with PBS and staining with 4′,6-diamidino2phenylindole (DAPI; Invitrogen). Images were acquired using a confocal microscope (BX60; Olympus, Tokyo, Japan).

### Western blotting analysis

To analyse PR expression, the cells were incubated in the presence of 10 nM/L β-oestradiol for 24 h. Cell lysate (10 μg) was separated on a 4–20% Mini-PROTEAN TGX Gel (BioRad, Hercules, CA, USA) and electrophoretically transferred to a polyvinylidene fluoride (PVDF) membrane (Trans-Blot Turbo Mini PVDF Transfer Packs; BioRad). The membrane was cut to an appropriate size prior to the following experimental procedures. First, the membranes were blocked in PVDF Blocking Reagent for Can Get Signal (Toyobo) and subsequently incubated with primary antibody in Can Get Signal Solution 1 for 12 h at 4 °C. Thereafter, membranes were washed with Tris buffer (20 mM/L Tris–HCl, pH 7.6; 137 mM/L NaCl; 0.1% Tween-20) and incubated with a secondary antibody in Can Get Signal Solution 2. For detection of PR, the primary antibody was rabbit anti-Progesterone Receptor A/B (D8Q2J; 1:5000 dilution), and the secondary antibody was ECL anti-rabbit IgG Horseradish Peroxidase-linked antibody (NA934, GE Healthcare, Chicago, IL, USA; 1:20,000 dilution). In addition, the anti-GAPDH antibody (5A12, Wako Co., Ltd., Tokyo, Japan; 1:50,000 dilution) and ECL anti-mouse IgG Horseradish Peroxidase-linked antibody (NA931, GE Healthcare; 1:50,000 dilution) were used as internal controls. Proteins were subsequently visualized using ECL Prime Western Blotting Detection Reagents (GE Healthcare) and imaged using a LAS-4000 Luminescent Image Analyzer (FUJIFILM Co., Tokyo, Japan). Images of PR and GAPDH staining were obtained using the same lane of the same gel, with an exposure time of 300 s for PR and 60 s for GAPDH.

## Supplementary Information

Supplementary Information.

## Data Availability

All RNA-seq data are available in the GEO under accession number GSE220806. Currently, token “etqnqiegljohraz” is issued for access.
