# Supplementary material for: Characteristic impairment of progesterone response in cultured cervical fibroblasts obtained from patients with refractory cervical insufficiency
Source: Sci Rep. 2023 Jul 20;13:11709. doi: 10.1038/s41598-023-37732-7 (PMC10359315; doi:10.1038/s41598-023-37732-7)

Characteristic impairment of progesterone response in cultured cervical fibroblasts obtained from patients with refractory cervical insufficiency

Authors

Yosuke Sugita[1], Yoshimitsu Kuwabara*[2], Shigeru Matsuda[1], Akira Katayama[1], Ichiro Manabe[3], Shunji Suzuki[2], Yumiko Oishi[1]

1. Department of Biochemistry and Molecular Biology, Nippon Medical School, 1-1-5 Sendagi, Bunkyo-ku, Tokyo, 113-8602, Japan.

2. Department of Obstetrics and Gynecology, Nippon Medical School, 1-1-5 Sendagi, Bunkyo-ku, Tokyo, 113-8602, Japan.

3. Department of Systems Medicine, Chiba University Graduate School of Medicine, 1-8-1 Inohana, Chuo-ku, Chiba, 260-8670, Japan.

Supplementary Table S1

genes upregulated by progesterone in RNA sequence

ZBTB16	RASL10B	TXNIP	EEF2K	TNS3	ERAP2
SPARCL1	CHAF1A	CCN4	TCTN2	PRRG1	ARHGEF25
CCL7	PSD	PER1	SERPINE1	ZHX3	SLC19A1
MOB3B	PTGER2	FAM53B	EDEM3	SMTN	ADAMTS1
DEPP1	CDC7	LPCAT1	MIS18BP1	USP28	PDE4A
ITGA10	BTBD11	MXD4	LINC00294	MIGA2	KIF1BP
FKBP5	CEBPD	SSH2	ACVRL1	GCNT1	ELK3
RASL11A	ABHD15	RIPOR2	HSPA12A	DENND6A	ST6GALNAC6
PDE7B	TARDBP	NFKBIA	TCEAL1	NACC2	FZD1
ZNF367	HSD11B1	FHOD1	PCNA	RAMP1	TAOK2
PRRG4	KLF9	SRRM3	SAP30L	FUT11	MOGS
PXYLP1	DCLRE1B	SAFB	RESF1	TNS2	SLC2A10
ORC1	JADE1	TBC1D4	ZCCHC24	ADCY6	NUP205
RGCC	C18orf54	CNPY4	EIF4EBP2	XYLT2	PHLDB1
SEC14L2	ELN	KLHL17	EXTL3	PRPS2	SLC27A4
DTL	DIXDC1	SLC26A2	PAPPA-AS1	KANK2	ELF1
FOXO1	PLEKHG3	C2CD2	NFIA	GPR161	NNMT
ARSI	HSPA2	C5orf30	MTMR4	OSBPL5	ZBTB47
HAND2	INTS8	HLX	TNFRSF1B	POLG	CLIC6
SLC40A1	MMD	PPARA	CERK	ATOH8	GLT8D1
EEF1AKMT4	NUP35	PSD4	NUP50	CBX6	APPL2
RARB	TMEM164	SLC29A1	PPP2R5D	SSBP3	MAVS
IL1R1	OTULINL	EIF4E2	ITSN1	GLUL	WDR6
SMIM3	LSM11	DNAJB4	FRMD8	MESD	DNM1L
MC1R	KCNK6	RAD9A	GSTT1	IP6K1	PDPN

Supplementary Table S2

genes downregulated by progesterone in RNA sequence

RAB33A	MKNK2	TRMT11	MAFF	BCAT1	ZNF770
CTH	PITX1	ODC1	TIMM44	PRAG1	PANK2
ATF3	OSGIN1	ARHGEF2	IVNS1ABP	BTF3L4	NR3C1
INHBE	SLC3A2	SPACA9	ZNF365	LOC646762	COX20
FGFBP3	GSTO2	ALPK2	DYRK3	SARS	FEM1C
IFIT2	SLC6A9	SLC30A1	ZNF23	TENT5C	JMJD1C
STC2	AFAP1L2	C10orf55	MSC	HECW2	MTHFD1L
CARS-AS1	C17orf107	SMOX	MMP16	COA5	ZFAND3
SLC7A11	ASNS	NCOA7	MARS	IFNGR1	IFIT5
ISLR2	AVPI1	ATP6V0A1	HIVEP1	TP53	ARMC9
DDIT4L	CZ1P-ASNS	IFRD1	TACC2	TBC1D19	NARS
GLI1	SNTB1	ARMC4	LONP1	TRIB1	BTBD10
MPP7	CREB5	RALA	SLC35F6	AFAP1	UTP25
AFF3	SLC38A1	ATF5	ZXDB	SHMT2	ZCCHC14
TNFSF15	GPT2	TES	ZCCHC8	AGPAT4	FOXP1
PCK2	KLF11	GRPEL2	NT5C3A	KDM6B	SLC16A7
SLC7A11-AS1	ICAM1	YARS	PIM3	FAS	CYLD
VLDLR	SEMA3D	KLF5	SLC7A1	FLRT2	CREB1
DDIT3	PSPH	APOL6	NUDT6	XPOT	DLC1
TNFAIP6	IFIT1	ADAMTS6	AKIRIN2	PIP4P2	NUB1
ARL4C	SCN9A	MIA2	TMEM267	INSIG1	CHD4
GDF15	ATP2B1-AS1	FGF2	HAX1	DCUN1D4	VPS37A
LURAP1L	ST7-AS1	AARS	SH3KBP1	RND3	ASAP2
MXD1	UHRF1BP1	CPEB2	NOTCH1	INO80	SPG11
IFIT3	HK2	GAS2L3	TNFRSF19	TRIM59	SMAD2
TUBE1	ETV5	RGS3	ALKBH3	PHGDH	ACSL3
E2F7	RCAN1	AP1AR	SH2D4A	ZNF462	OSMR
CARS	FBXO10	SUPV3L1	WDFY2	AP4E1	CAMSAP2
SESN2	TRIM16	SEPHS2	ETS1	BACH1	UHRF2
KCNA3	MTHFD2	DUS4L	TCEA1	ECD	TAOK3
GOT1	GCLC	RHBDD1	SERINC5	WDR74	PPP1R15B
RASSF2	ATP6V0D2	ANKRD11	TULP4	ACVR1	AFF4
CNIH3	SYNE1	USP54	HSPA13	USP32	NDFIP2
ZBED3	BBC3	EPB41	ZNF697	BABAM2	KDM5C
KRT34	CBX4	RMND1	DUSP12	DDR2	FBXO11
TMEM268	PLAU	RNF41	STK40	FAM155A	BCLAF1
NR1D2	LRRC49	GS1-124K5.11	EPRS	CEP57	KIRREL1
NIBAN1	TMEM154	WARS	ZBTB21	NFE2L2	
PPP1R15A	IRF1	VDR	DDX19B	ITSN2	
PSAT1	LARP6	TSPYL4	LIPA	ABL2	

Supplementary Figure S1

Full-length western blotting gel shown in Fig.3.

Western blotting was carried out using 10 µg of protein extracted from cells cultured without or with E2 (10 nmol//L) PR-A:90kDa, PR-B:118kDa. Images of PR and GAPDH staining were obtained using the same lane of the same gel, with an exposure time of 300 s for PR and 60 s for GAPDH.

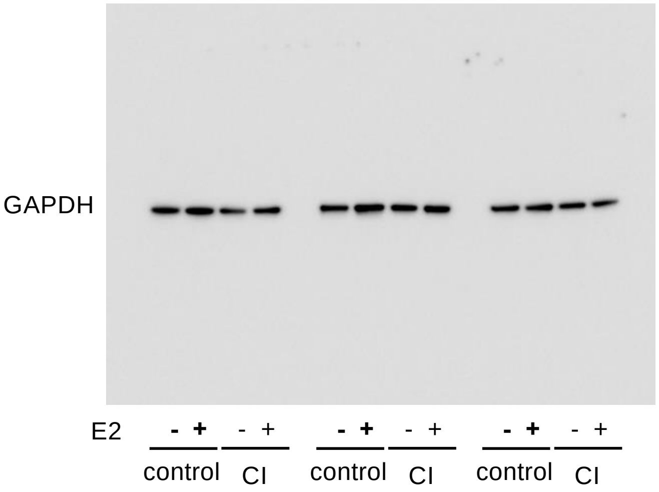

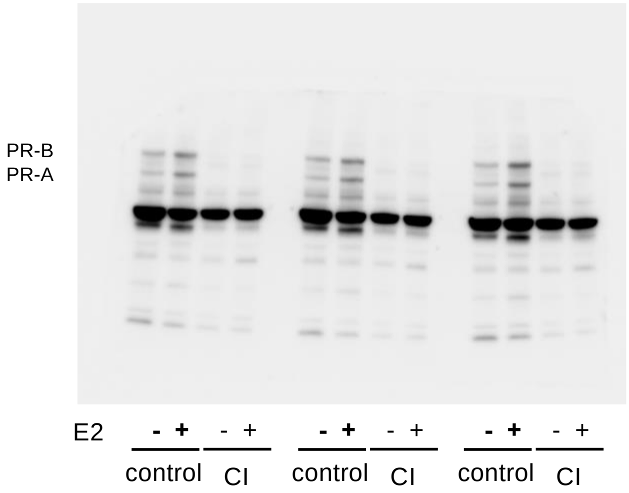

Supplementary Figure S2

Results of RT-PCR analysis on the expression changes of cervical ripening-related genes in response to P4 stimulation

The cells cultured from control1 and CI1 were treated with 1μM P4 or the vehicle (ethanol) for 6 h according to the experimental design described in the text. RNA was extracted from these cells, and reverse-transcribed cDNA was generated for RT-PCR analysis. Genes that are known to exhibit expression changes in the cervical tissue along with cervical ripening were targeted in this analysis. The expression levels of each gene were compared to the internal control (GAPDH) and the ratios were plotted. Genes showed no significant changes in response to P4. Data are presented as the mean ±SEM and analysed using a ratio paired t-test. ns: not significant.

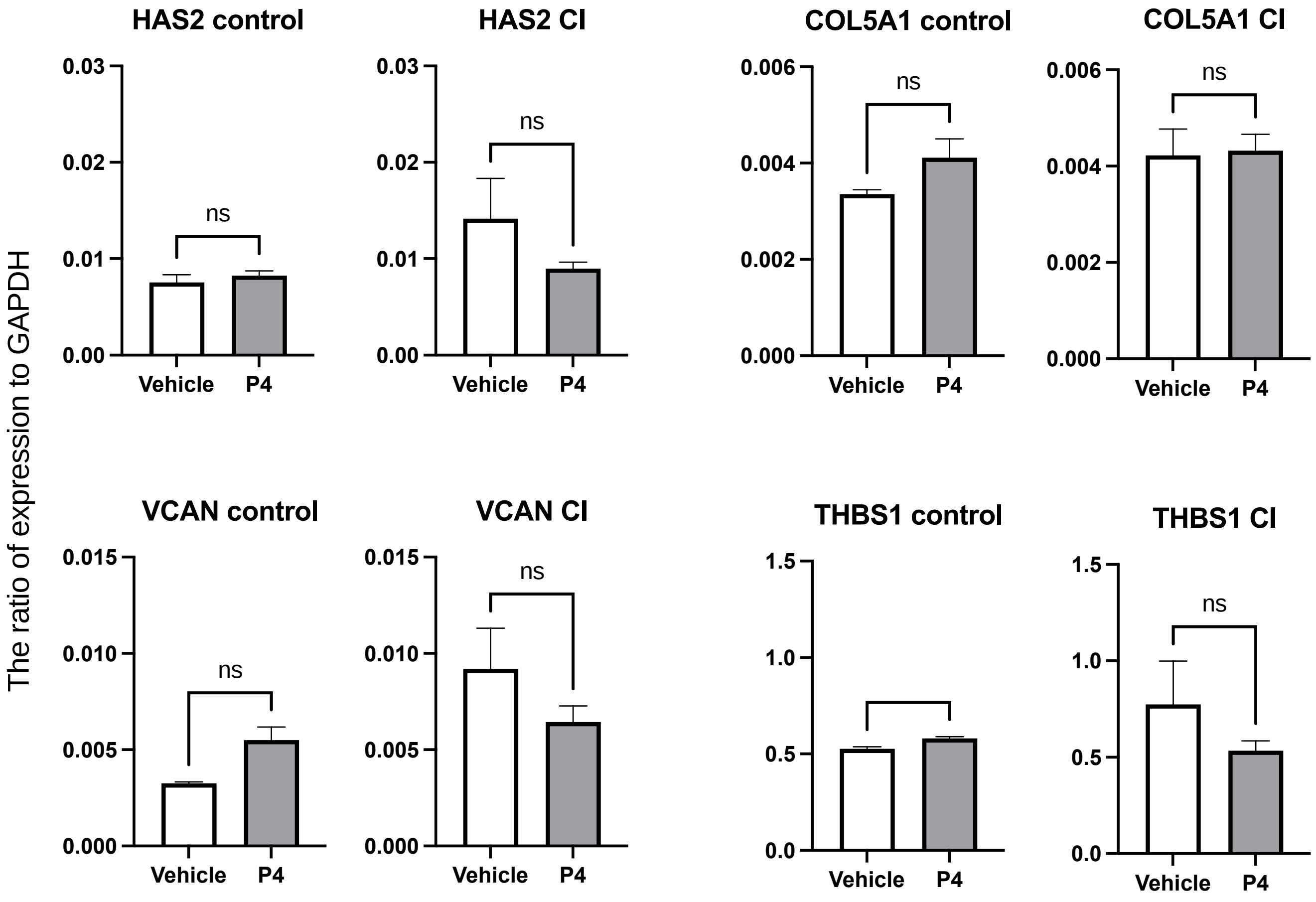

Supplementary Figure S3

Comparison of expression of steroid receptor genes between control and CI using RT-PCR

Cells were collected from control (n=3) and CI (n=3) samples, which were cultured under the presence of 10 nmol/L beta-estradiol as described in the text. RNA was extracted from these cells, and reverse-transcribed cDNA was generated for the comparison of estrogen receptor (ESR1) and glucocorticoid receptor (NR3C1) expression using RT-PCR. The expression levels of each gene were compared to the internal control (GAPDH) and the ratios were plotted. No significant differences were observed in the expression of ESR1 and NR3C1 between control and CI. Data are presented as the mean ±SEM and analysed using a t-test. ns: not significant.

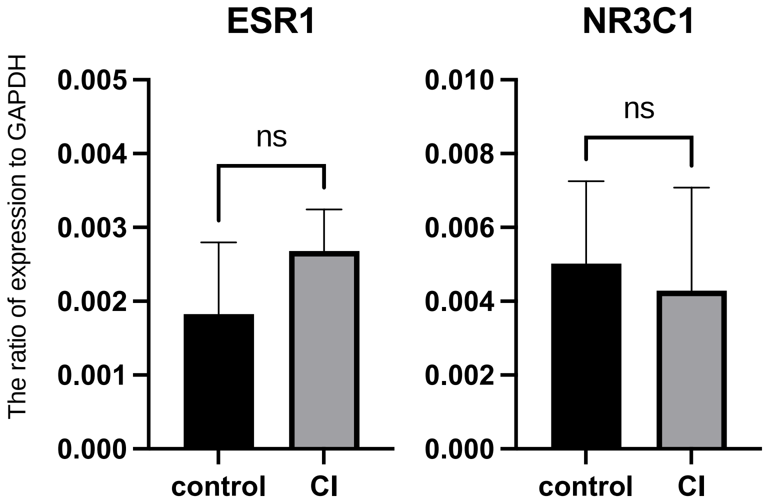

Supplement: Supplementary file 1 — Supplementary Information. [file 41598_2023_37732_MOESM1_ESM.pdf]
